# Efficacy of Dashmool Vasti as an adjuvant therapy with the standard of care (modern + physiotherapy) in the rehabilitation of stroke as compared to standard of care – a clinical trial protocol

**DOI:** 10.12688/f1000research.141553.3

**Published:** 2024-04-10

**Authors:** Punam Sawarkar, Vaishali Kuchewar, Gaurav Sawarkar, Irshad Qureshi

**Affiliations:** 1 Mahatma Gandhi Ayurved College Hospital and Research Centre, Datta Meghe Institute of Higher Education and Research, Wardha, Maharashtra, 442001, India; 2 Ravi Nair Physiotherapy College, Datta Meghe Institute of Higher Education and Research, Wardha, Maharashtra, 442001, India

**Keywords:** Stroke, Pakshaghata, Panchkarama, Niruha Vasti, Dashmoola, Adjuvant, Physiotherapy, MRP, FES

## Abstract

**Background:**

Stroke is ischemia and neurological dysfunction caused by acute brain circulation loss. It causes acute localized neurological abnormalities such as weakness, sensory deficit, or language issues that require long-term treatment. These deficiencies harm the patient and their family psychologically, socially, and economically. Thus, combination treatment can rapidly rehabilitate such patients. Detoxification methods like Ayurvedic medicated enema help stroke pathophysiology. Physical modalities in physiotherapy have been shown to facilitate normal movement and function on the stroke patient’s affected side, increasing independence with everyday duties. A stroke patient may benefit from *Dashmoola Niruha Basti*, Function Electrical Stimulation (FES), and Motor Relearning Programme (MRP).

**Aim & Objectives:**

This study compares the adjuvant role of *Dashmoola Basti* with MRP and FES in stroke recovery. The main goals of this study are to assess and compare the adjuvant role of *Dashmoola Basti* with standard control over sensorimotor function of lower extremities, static and dynamic balance in stroke patients; gait parameters; resistance experienced during passive range of motion; quality of life of patients; Barthel Index; Modified Ashworth Scale; and Fuglmeyer assessment, Single Limb Stance Test, Functional Reach Test.

**Methods:**

A total of 40 patients will be enrolled and divided randomly into two equal groups. In Group A (control), standard treatment (modern + physiotherapy) will be prescribed for one month. In Group B (interventional group), *Dashmoola Basti* will be added to the aforementioned standard treatment for one month.

**Expected results:**

Improvement in Fuglmeyer assessment, Single Limb Stance Test, Functional Reach Test, quality of Life of Patients, Barthel Index, Modified Ashworth scale, and National Institute of Health (NIH) stroke-scale-score will be observed and recorded.

**Conclusions:**

Results and conclusions will be derived according to the data collected in case record form and assessment sheets filled at baseline and follow-up visits.

**Trial registration:**

CTRI/2021/10/037445 dated 21.10.2021.

## Introduction

In clinical practice, stroke has been very notorious for impeding the continuity of life and day-to-day activities. It is the most common example of such a crippling disorder. On extensive review of contemporary literature, it is observed that, despite massive worldwide efforts in this science to rectify early results in reducing the risk of death or disability, it is disappointing. ^1^ Partial recovery is obtained after a particularly long time. Moreover, it is observed that there are certain limitations, contra-indications, or side effects of currently established treatment modalities for stroke. ^2^

Considering the complicated and critical nature of the disease, i.e., stroke, it becomes imperative to search for safe, promising, and effective treatment modalities in alternative sciences for early rehabilitation purposes for stroke. Though most of the studies suggest that the individual use of *Panchakarma* therapy, external oleation ( *Bahya Snehana*), sudation therapy ( *Swedana*) along with medicated enema ( *Basti*) and conventional physiotherapy, i.e., Motor Relearning Programme (MRP), and the combination of electrical modality with Functional Electrical Stimulation (FES) shows promising results in the rehabilitation of patients with stroke.

However, no study has been carried out to assess their combined effects on the therapeutic outcome of the rehabilitation of such patients. Moreover, the research is also required to determine the MRP and FES combined effect of supporting stroke patients for improving knee extension and gait parameters. Therefore, this study is proposed to generate clinical evidence showing excellent post-stroke recovery within a short time in restoring gross and fine movements to the maximum extent quicker.

The study aims to analyze and compare the adjuvant role of *Dashmoola Basti* with standard treatment in stroke rehabilitation. In addition, the efficacy of physiotherapy (MRP and FES) in rehabilitating stroke patients will be assessed. The study’s goal is to assess and compare the adjuvant role of *Dashmoola Basti* with standard control over sensorimotor function of lower extremities in stroke patients using the Fugl-Meyer assessment, as well as to assess and compare the adjuvant role of *Dashmoola Basti* with standard control over static and dynamic balance in stroke patients using the Single Limb Stance Test and Functional Reach Test, respectively. The secondary goal is to evaluate and compare the adjuvant role of *Dashmoola Basti* with standard control over gait parameters (distance and time parameters), the Barthel Index, resistance experienced during passive range of motion based on the modified Ashworth scale, and the National Institute of Health (NIH) stroke-scale-score in stroke patients.

## Methods

### Ethical considerations

IEC clearance is taken from the Institutional Ethical Committee, Datta Meghe Institute of Higher Education and Research (DMIHER), Wardha. IEC certificate obtained: Ref No. DMIHER (DU)/IEC/2021/279 dated 15.04.2021. The project has been registered with Clinical Trials Registry India (CTRI) (registration number CTRI/2021/10/037445) dated 21.10.2021).

Written informed consent will be taken from the patient before starting the study. During the study, the confidentiality of each patient will be maintained.

### Study design

It is an interventional study, it is a superiority clinical trial, i.e., randomized reference standard control open-labeled two-arm comparative clinical trial.

### Sample size

Due to practical considerations such as cost, patient inconvenience, judgments not to proceed with an investigation or a lengthy study duration, the number of participants in the study is restricted. So for the phase-I trial, 20 sample size is considered for each group. ^3^

### Recruitment

Daily visit to the stroke inpatient ward is planned at Acharya Vinoba Bhave Rural Hospital, and if the patient is willing to enroll in the said study can be recruited for the study after written informed consent. Patients will be added one at a time until the target sample size is reached.

### Setting

Locus of the study: out patient department and inpatient department of Panchakarma & Kayachikitsa department, Mahatma Gandhi Ayurveda College Hospital and Research Centre (MGACH&RC), Salod (Hirapur) Wardha, Maharashtra. & Department of Neuro physiotherapy, Ravi Nair Physiotherapy College, Sawangi, Wardha Maharashtra, Datta Meghe Institute of Higher Education and Research, Wardha, Maharashtra.

Study setting: The study will be conducted in *Panchakarma* OPD & IPD, Mahatma Gandhi Ayurveda College Hospital and Research Centre (MGACH&RC), Salod (Hirapur) Wardha, Maharashtra.

Patients will be recruited after approval from Clinical Trials Registry - India (CTRI), and the exposure period for treatment will be one month. The follow-up will be conducted on the 60 ^th^ and 90 ^th^ day of treatment.

Criteria for discontinuing or modifying: if patients are willing to quit in between they will be allowed to quit and will be replaced; if the patient develops an acute illness during the trial, which may hamper the study and withdrawn patients will be replaced.

### Participants

Diagnosed stroke cases will be enrolled in the study with the computerized randomization method.

### Eligibility criteria

The inclusion criteria for the study are the patients with the first stroke diagnosed with CT/MRI (thrombolytic stroke only) without other neurological deficits but < six months of onset, patients diagnosed as stroke with ICD code 2020 ICD-10-CM Diagnosis Code I69.351 patients between 45 to 60 years of age irrespective of gender/occupation and socio-economic status. Patients with controlled hypertension and Non-insulin dependent Diabetes Mellitus (NIDDM). Patient with ankle dorsiflexion stage 1 to 2 and ankle plantar flexors spasticity 1+ on. Patients are willing to give informed consent and ready to follow simple instructions.

The exclusion criteria for the study are the patients having a thrombolytic stroke with onset for > six months, subjects with complications such as uncontrolled metabolic disorders and severe systemic disorders, e.g., renal or cardiac failure, altered sensorium, or coma. Those who are on tube feeding or intravenous fluid therapy. The patients of stroke with a history of trauma ( *Abhighatajanya*), onset due to intracranial space-occupying lesions, post-surgical or postpartum complication, degenerative disorders of the brain or intracranial infectious disease or hemorrhagic nature. The patients with specific medical and psychological contraindications for electrical stimulation Brooks, ^4^ patients with visual or auditory difficulties, anti-anxiolytic treatments. Pregnant women and lactating mothers will also be excluded. Patients contraindicated for *Basti & Swedana* therapy and patients with fixed ankle or foot contracture, pacemakers, and a metal plate in the lower limb will be excluded.

### Interventions

For group A, standard control modern treatment (Anti-thrombotic treatment, e.g. Tab. Ecosprin 75/150 mg OD and Tab. Notropril 800 mg TDS) will be given along with physiotherapy. The physiotherapy sitting for motor relearning program and functional stimulation will be given for 55 mins per sitting, five days weekly for one month.

For group B, standard control modern treatment will be given along with Ayurveda Panchakarma and physiotherapy. In this case, gentle massage with *Dashmoola Taila* + *Nadi Sweda* with *Dashmoola Kwatha* + medicated enema (alternate regime of *Dashamoola Niruha Basti* with *Anuvasana Basti* with *Dashmoola Taila* will be prescribed for the treatment. The alternative regime of medicated enema i.e., *Anuvasana Basti* (oil enema) and *Dashamoola Niruha Basti* (decoction enema) will be given on odd and even days respectively for one month, and physiotherapy for five days weekly for one month.

**Figure 1.**
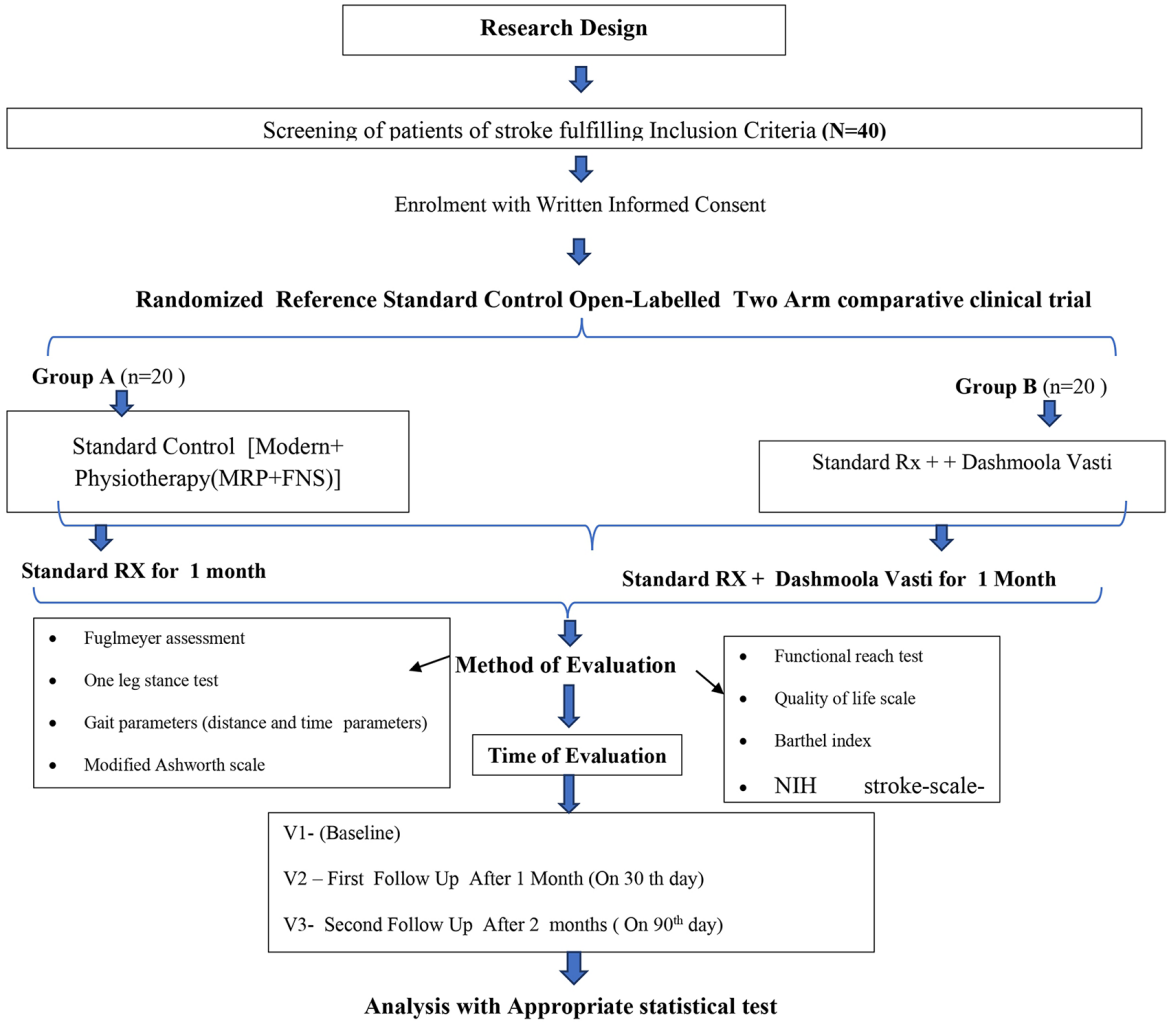
Study design including grouping, intervention, assessment plan and follow-up. *FES – Function Electrical Stimulation, MRP – Motor Relearning Programme, NIH – National Institutes of Health.

The study’s methodology is mentioned in Table 1 and the flowchart of the study design or methodology is given in Figure 1.

**Table 1.** Methodology of the study.

Group	Sample size	Intervention	Type of Treatment	Duration of treatment	Total duration	Follow-up
**A**	20	Standard Control	Modern + Physiotherapy (MRP+FES)	For Physiotherapy sitting (55 mins/sitting)	Physiotherapy (Five days weekly for one month)	on 60 ^th^ & 90 ^th^ Day
**B**	20	Standard Control + *Panchakarma*	Modern + Physiotherapy (MRP+FES) Gentle massage with *Dashmoola Taila* + *Nadi Sweda* with *Dashmoola Kwatha* + Medicated Enema (Alternate regime of *Dashamoola Niruha Basti* with *Anuvasana Basti* with *Dashmoola Taila*	• Same as group A+ *Dashmoola Basti* Details of *Dashmoola Basti* Are as follows: • Local *Snehana*-10 mins • Local *Swedana*-10 mins or till attainment of *Samyak Swinna Lakshana*, whichever is earlier.	• Panchakarma for complete one month (Day 1 ^st^ – Day 30 ^th^) • Physiotherapy for Five days weekly for one month	on 60 ^th^ & 90 ^th^ Day

* FES – Function Electrical Stimulation, MRP – Motor Relearning Programme.

### Assessment criteria

The assessment criteria will be the Fugl-Meyer assessment, one-leg stance test, gait parameters (distance and time parameters), modified Ashworth scale, functional reach test, quality of life scale, Barthel index, NIH stroke scale score (NIHSS). ^5^^,^^6^

### Variables to be measured

Improvements in the following assessment variables are expected as primary outcomes: Fugl-Meyer assessment for sensory-motor function of lower extremities, one-leg stance test, functional reach test for static and dynamic balance in patients, quality of life scale and Barthel index independent living of patients with Stroke, resistance experienced during passive range of motion based measured on Modified Ashworth scale NIHSS for improvements in clinical features of stroke are expected to be measured. ^7^

### Participant timeline

• For physiotherapy (five days weekly for one month)
• *Karma Basti* (alternate regime of *Niruha Basti* with *Dashmoola Kwatha* and *Anuvasana Basti* with *Dashamoola* oil for complete one month, i.e., Day 1 ^st^–Day 30 ^th^)

Time schedule of enrolment: Patients with the first Stroke diagnosed with CT/MRI (thrombolytic Stroke only) without other neurological deficits but <6 months of onset will be enrolled for the study.

Interventions (including any run-ins and washouts): There are no washout periods; the intervention of Panchakarma treatment and Physiotherapy sitting for 30 days.

Assessments and visits for participants: Assessment will be carried out at baseline level and after the intervention, i.e., on the 30 ^th^ day, then follow-up visits on the 60 ^th^ and 90 ^th^ day.

### Assignment of interventions (for controlled trials)

Patients will be recruited by simple randomization through the computer-generated table. The principal investigator (PI) and co-investigator (CO-I) will allocate and enroll the patient. Using computer-generated random numbers, the researcher creates random allocation cards. The researchers will keep the original randomly assigned sequences in a safe place and use a different copy to sign up the patients.

The envelopes will be marked with serial numbers on the outside. The date, time, patient ID, post-procedure results, and other required details will be on the envelope.

### Data collection, management, and analysis methods

For retention of patients, appointment reminders will be scheduled, and question-answering sessions will be planned. The complete follow-up will be mentioned in the patient file, and if patients are required to withdraw or discontinue, the patient file will be closed and maintained for the record purposes.

Observations will be made after the completion of the study, according to the data collected with the help of the following:

I. Case registration form with detailed history and examinations
II. Follow-up assessment proforma

The principal investigator and co-investigator will do data monitoring and coding. Data entry will be done in soft copy and case record form in hard copy. The values will be verified twice before submission in soft copy. Each file of the patient is secured with a specific patient code.

The data obtained will be calculated using the Student’s Paired ‘t’-test and Unpaired ‘t’ test for objective variables with SPSS statistical software.

*To verify the significance of the results*

Improvements in Fugl-Meyer assessment-35%, one leg stance test-20%, functional reach test-35%, quality of life scale-25%, Barthel index-25% and NIH stroke-scale-score-20% will be considered as significant.

## Discussion

Cerebrovascular accidents (CVA) can be correlated with *Pakshaghata* in Ayurveda. Its pathophysiology evolves in *Shira* (head). In Ayurveda, *Panchakarma* is considered the most effective regime to break the pathogenesis of the disease from the root and avoid its recurrence. *Snehana*, *Swedana*, including *Basti*, is the ultimate treatment modality for *Pakshaghata*. According to Acharya Sushruta, *Basti* provides more than half of the treatment of disorders with chronic and deeply embedded pathology and is primarily effective for conditions arising from the morbid *Vata Dosha*. ^8^^,^^9^

It is the most important as it drastically expels the vitiated *Vata* responsible for the movements of all *Dosha, Dhatu*, and *Mala* within the body. *Niruha Basti* serves the purpose of the elimination of vitiated *Vata Dosha*. As *Shosha* of *Sira* and *Snayu*, which is the most critical event in the *Samprapti* of *Pakshaghata*, the *Bruhana* effect induced by *Anuvasana Basti*, which pacifies *Vata*. Being an obstinate *Vata* disorder, *Pakshaghata* demands a pioneering treatment of *Vata*, i.e., *Basti*, especially *Shodhana* or *Bruhana Basti*, for a more extended period. Therefore, *Basti* is considered one of the foremost treatments for *Pakshaghata*, capable of eliminating Doshas from the body. ^10^^,^^11^ It sustains life by maintaining the harmony between *Dosha, Dhatu*, and *Mala* in the body. ^12^^,^^13^

Among various types of *Niruha Basti Dravyas*, *Dashamula*, i.e., a combination of the 10 drugs, is the best *Tridoshahara*, especially has *Vatahara* property; therefore, it is highly efficacious for *Vata* predominant disorders. *Dashmoola Niruha Basti* and *Anuvasana Basti* with *Dashmoola* Oil are considered very effective, providing additional benefits of *Shodhana* and *Rasayana*, which are expected in *Pakshaghata*. ^14^^–^^16^

Due to this devastating and refractory nature of *Pakshaghata*, a minimum course of three months of appropriate treatment is recommended. Therefore, among detoxification procedures, *Karma Basti* (course of 30 *Basti* having a regime of alternate 18 *Anuvasana Basti* and 12 *Niruha Basti*) with *Snehana* and *Swedana* are selected for this study. ^17^^–^^20^

Local *Snehana* and *Swedana* is the mandatory pre-procedural protocol before administering both *Niruha* and *Anuvasana Basti* that is helpful to achieve expected proper procedural symptoms (i.e., *Samyak Niruha* and *Anuvasana Basti Lakshana*). ^21^ Moreover, it also increases the therapeutic outcome of these therapies by improving blood circulation in that local region.

As local massage nourishes the muscular tissue and fomentation with *Dashmool* decoction, massage instantaneously relieves the extremities’ stiffness, especially muscular tissues. Both these procedures also play an important role in enhancing the speed of rehabilitation of the stroke, resulting in a speedy recovery in cognitive functions hampered in it. According to Ayurveda, vitiation of *Vata* is the most important factor responsible for inducing impairment of cognitive functions and musculoskeletal movements. ^22^ Both procedures used *Dashmoola* oil and *Dashmoola* decoction, respectively, to pacify vitiated *Vata* due to their *Snigdha* & *Ushna Guna*. Moreover, both improve the patient’s gait and reduce the dependency of the affected individual on relatives. ^23^^,^^24^ Local massage and fomentation with *Vata* pacifying medicines improve the patient’s quality of life by building confidence and contributing to inducing expected positive outcomes. ^25^

On the other hand, the efficacy of physiotherapy, especially of the “Motor Relearning Program” (MRP) and “Functional Electrical Stimulation” (FES) in the early rehabilitation of the stroke can be justified as follows:

Motor learning theory underpins the Motor Relearning Programme (MRP). Carr and Shepherd claimed that motor control training necessitates anticipatory acts as well as continuing exercise. The motor relearning program is useful for improving a group of post-stroke patients’ balance function and functional performance. It consists of four distinct steps, i.e., to analyze the task and practice the missing component and task with a transference of training. Eight studies suggested that MRP planned for five weeks showed better improvement in functional performance on self-care, instrumental activities of daily living, and integration into the community hospital. ^26^

The FES is based on electrical stimulation to the peripheral nerves that innervate the paralyzed muscle to generate action potentials in motor neurons, propagating towards the power and causing its contraction. According to John E.R. 1999 *et al.* and Niu *et al.*, 2019, it is an excellent recovery tool for motor functions hampered in post-stroke conditions.

Both MRP and FES are quite useful for regaining full mobility in such patients, enhancing muscle strength and endurance, expanding movement capacity, reducing atrophy, increasing walking speed, reducing spasticity, and relieving pain. This technique is quite effective in improving the swing phase of the gait, correcting a foot drop, and decreasing the energy expenditure during walking by restoring ankle dorsiflexion affected in the patient with stroke. ^27^^,^^28^

In a nutshell, all-composite treatment modalities induce restoration of gross and fine movements, increase gait speed to the maximum extent quicker, decrease falls, and improve he patient’s quality of life.

## Conclusion

Shreds of evidence generated from the current study may prove the positive benefits of the use of integrative approach of *Dashamool Niruha Basti* in *Panchakarma* added with MRP and FES in physiotherapy, to improve gait and speed of movement, decrease the rate of fall while standing or walking, and improves quality of life of patients having stroke. This treatment protocol may become an effective tool in the rehabilitation of such patients, which may broaden the scope of Ayurveda in neurology.

### Glossary

*Abhighatajanya* – Trauma

*Anuvasana Basti* – Oil enema

*Bahya Snehana* – External oleation

*Dashmool* – Combination of 10 Ayurveda drugs

*Dhatu* – Body tissues

*Dosha* – Bio-humours

*Karma Basti* – Alternate regime of decoction enema & medicated oil enema for complete one month

*Kayachikitsa* department – Medicine department

*Mala* – Body toxins

*Niruha Basti* – Decoction enema

*Niruha Basti Dravyas* – Decoction enema content

*Pakshaghata* – Paralysis

*Panchakarma* department – Purification Putative treatment department

*Panchkarama* – Purification putative treatment

*Rasayana* – *Rejuvenation*

*Samprapti* – Pathophysiology

*Shira* – Head

*Shodhana* – Purification

*Snigdha Guna* – Unctuousness quality

*Swedana* – Sudation therapy

*Tridoshahara* – Bio-humour Alleviator

*Ushna Guna* – Hot quality

*Basti* – Medicated Enema

*Vata Dosha* – Air entity in the body

*Vatahara* – Air entity pacifier

## Data Availability

No data are associated with this article. Figshare: SPIRIT checklist for ‘Efficacy of *Dashmool Basti* as an adjuvant therapy with the standard of care (modern + physiotherapy) in the rehabilitation of stroke as compared to standard of care – a clinical trial protocol’. https://doi.org/10.6084/m9.figshare.24488059. Data are available under the terms of the Creative Commons Zero “No rights reserved” data waiver (CC0 1.0 Public domain dedication).
