# Supplementary material for: Efficacy of Dashmool Vasti as an adjuvant therapy with the standard of care (modern + physiotherapy) in the rehabilitation of stroke as compared to standard of care – a clinical trial protocol
Source: F1000Res. 2024 Apr 10;12:1589. Originally published 2023 Dec 14. [Version 3] doi: 10.12688/f1000research.141553.3 (PMC11016165; doi:10.12688/f1000research.141553.3)
Supplement: Peer review history

# Reviewer report: Reviewer response for version 3

**DOI:** 10.5256/f1000research.164455.r265099

**Published:** 2024-04-13

**Authors:** Aishwarya Ashish Joglekar, Sumedh Joshi

**Affiliations:** 1 Samhita Siddhant, All India Institute of Ayurveda, New Delhi, New Delhi, India; 2 Dravyaguna, All India Institute of Ayurveda, New Delhi, New Delhi, India

I have no further comments to make. Great Effort on the part of the Authors

Is the study design appropriate for the research question?

Yes

Is the rationale for, and objectives of, the study clearly described?

Yes

Are sufficient details of the methods provided to allow replication by others?

Yes

Are the datasets clearly presented in a useable and accessible format?

Yes

Reviewer Expertise:

Ayurveda, Endocrinology , Musculoskeletal system, Preventive health

We confirm that we have read this submission and believe that we have an appropriate level of expertise to confirm that it is of an acceptable scientific standard.

# Reviewer report: Reviewer response for version 2

**DOI:** 10.5256/f1000research.162587.r257309

**Published:** 2024-03-27

**Authors:** Aishwarya Ashish Joglekar, Sumedh Joshi

**Affiliations:** 1 Samhita Siddhant, All India Institute of Ayurveda, New Delhi, New Delhi, India; 2 Dravyaguna, All India Institute of Ayurveda, New Delhi, New Delhi, India

This is a well written article considering important protocol for management of Pakshaghata as per principles of Ayurveda. Few modifications are however required which are communicated earlier- 

**In the sentence - "He will keep the original random allocation sequences in a secure third location and work with an alternate copy"- Grammatical Correction is required**

**Correct the sentence- Acharya Sushruta states that *Basti* contributes to more than half to the  management of diseases having chronic and deeply rooted pathology and is primarily helpful for conditions originating from the morbid *Vata Dosha*.**

**Give reference - Local *Snehana* and *Swedana* is the mandatory pre-procedural protocol before administering both *Niruha* and *Anuvasana Basti* and is helpful to achieve expected proper procedural symptoms (i.e., *Samyak Niruha* and *Anuvasana Basti Lakshana*).**

 massage **quickly** relieves the extremities’ stiffness, especially muscular tissues. Use term instantaneously instead of quickly

**Give reference for the statement- **As local massage nourishes the muscular tissue and fomentation with *Dashmool* decoction, massage quickly relieves the extremities’ stiffness, especially muscular tissues. Both these procedures also play an important role in enhancing the speed of rehabilitation of the stroke, resulting in a speedy recovery in cognitive functions hampered in it. According to Ayurveda, vitiation of *Vata* is the most important factor responsible for inducing impairment of cognitive functions and musculoskeletal movements.

*Bahya Snehana* – Especially external oleation omit the word especially

 Tridoshahara-  Bio-humour Alleviator

Is the study design appropriate for the research question?

Yes

Is the rationale for, and objectives of, the study clearly described?

Yes

Are sufficient details of the methods provided to allow replication by others?

Yes

Are the datasets clearly presented in a useable and accessible format?

Yes

Reviewer Expertise:

Ayurveda, Endocrinology , Musculoskeletal system, Preventive health

We confirm that we have read this submission and believe that we have an appropriate level of expertise to confirm that it is of an acceptable scientific standard, however we have significant reservations, as outlined above.

# Reviewer report: Reviewer response for version 2

**DOI:** 10.5256/f1000research.162587.r247406

**Published:** 2024-02-28

**Authors:** Neha Tank

**Affiliations:** 1 Department of Panchkarma, Institute of Teaching and Teaching in Ayurveda (Institute of national Importance) Ministry of Ayush, Jamnagar, Gujarat, India

I have no further comments.

Is the study design appropriate for the research question?

Yes

Is the rationale for, and objectives of, the study clearly described?

Yes

Are sufficient details of the methods provided to allow replication by others?

Partly

Are the datasets clearly presented in a useable and accessible format?

Partly

Reviewer Expertise:

Panchakarma, Autoimmune diseases, Neurological diseases, Osteomuscular diseases, degenerative diseases, Stress and allergies

I confirm that I have read this submission and believe that I have an appropriate level of expertise to confirm that it is of an acceptable scientific standard.

# Reviewer report: Reviewer response for version 1

**DOI:** 10.5256/f1000research.155010.r231867

**Published:** 2024-01-22

**Authors:** Neha Tank

**Affiliations:** 1 Department of Panchkarma, Institute of Teaching and Teaching in Ayurveda (Institute of national Importance) Ministry of Ayush, Jamnagar, Gujarat, India

grammatical corrections required.

 Spell check e.g. Kwatha not Quath 

 Basti not Vasti

 Non English words (Sanskrit) may be in Italic  

 Assessment criteria is not submitted, study results in terms of demographic data, clinical data, blood report changes and statistical analysis are not submitted.

 duration of basti is too long for any stroke patient.

 Any findings related to adverse effect/complication of Basti?

 study details regarding effect of Basti is not submitted

Is the study design appropriate for the research question?

Yes

Is the rationale for, and objectives of, the study clearly described?

Yes

Are sufficient details of the methods provided to allow replication by others?

Partly

Are the datasets clearly presented in a useable and accessible format?

Partly

Reviewer Expertise:

Panchakarma, Autoimmune diseases, Neurological diseases, Osteomuscular diseases, degenerative diseases, Stress and allergies

I confirm that I have read this submission and believe that I have an appropriate level of expertise to confirm that it is of an acceptable scientific standard, however I have significant reservations, as outlined above.

# Response

**Published:** 2024-02-11

**Authors:** punam sawarkar

Dear Madam,

 The updated version of the manuscript incorporates helpful comments, including correcting grammatical errors; all Ayurveda terms are made italic, Kwatha and Basti's spelling has been corrected, and Assessment criteria are specified in the text.

 Because the current manuscript is a PROTOCOL instead of an original article, so some suggestions of respected reviewers can not be incorporated.  (For example, study results regarding demographic data, clinical data, blood report changes, and statistical analysis are not submitted, any findings relating to adverse effects/complications of Basti?, and study details addressing the effect of Basti are not submitted.)

 According to reviewers, the duration of Basti is too long for any stroke patient; nonetheless, the author submits that due to the devastating and refractory character of Pakshaghata, a minimum term of three months of proper treatment is advised. As a result, Karma Basti (a 30-day detoxification regimen consisting of 18 Anuvasana Basti and 12 Niruha Basti) with Snehana and Swedana was chosen for this study.
